# Advances in the Control of the Helminthosis in Domestic Animals

**DOI:** 10.3390/pathogens12091107

**Published:** 2023-08-30

**Authors:** Jackson Victor de Araújo

**Affiliations:** Departamento de Veterinária, Universidade Federal de Viçosa (UFV), Viçosa 36570-000, Brazil; jvictor@ufv.br

The damage caused by parasitic worms is related to delays in production, the cost of prophylactic and curative treatments and, in extreme cases, the death of animals. While in developed countries spending on control costs is significant, in developing countries, parasitic diseases cause losses by reducing production and restricting the breeding of animals with reduced susceptibility to parasitosis, but with low production performance. The lack of this information can lead to the inappropriate use of anthelmintic treatments related to the rapid development of resistance and translated into an increase in clinical cases and production losses. The problems related to resistance and ecotoxicity emphasize the need to implement integrated parasite control programmes that ensure the health and safety of living organisms through strategic treatments based on epidemiology, the elimination of unnecessary deworming and the use of sanitation The amounts ingested and the characteristics of each organism vary depending on the chemical group. On the other hand, the world’s human population is increasingly demanding healthier, residue-free food that is produced in a way that preserves the environment. Helminthosis represents a global problem that is attributed not only to financial losses, but also to losses caused by damage to animal health. To overcome this problem, the most common behavior of owners and breeders is to resort to the use of anthelmintic chemicals. However, this alternative has often presented unsatisfactory results, since the occurrence of the development of resistance by parasites to commercially available antiparasitic drugs has been reported frequently in several countries. Changes in consumer perspective regarding animal welfare in production and sustainability have conquered a significant share of the market as a result of demand for chemical-free products. Among the advances for the control of helminthosis, we have the biological control, vaccines, resistant breeds, nanotechnology and even new anthelmintics, whether chemical or phytotherapeutic, that are necessary for the dynamics of the control of helminthosis.

The aim of this Special Issue was to present new developments in the control of helminthiases in domestic animals. Research in this area has been reduced over time compared to entomology and protozoology, often due to the control of experimental infections and even the control of bodies and ethics committees in animal experimentation, mainly in the sacrifice of animals to see the parasite load of worms in the animals, as we are dealing with endoparasites. The various studies presented here deal with the following aspects:

**Biocontrol**. Ribeiro et al. verified the predatory capacity of *M. expansa* eggs with an experimental formulation containing *Duddingtonia flagrans* and *Pochonia* [1]*. Chlamydosporia* fungi has the potential for application in integrated helminth control programs, with *P. chlamydosporia* being a species with ovicidal effect. Viña et al. created a new formulation with *D. flagrans* and deconstrued an efficient solution to decrease the risk of infection among dogs maintained in shelters, which is recommended [2]. Fonseca et al., with a formulation with fungus *Pochonia chlamydosporia*, concluded that the rapid transition from egg to larvae stage was not affected by fungus *Pochonia chlamydosporia*; therefore, the fungal formulation containing *Pochonia chlamydosporia* was not efficient in the biological control of bovine gastrointestinal nematodes [3]. The results of Luca et al. indicated an ovicidal action of *Pythium oligandrum*, supporting the prospects of its use in the decontamination methods of various surfaces or environments where ascarid eggs from carnivores are found [4]. **Diagnostic.** Reyes-Guerrero et al. mentioned that their study deepens our knowledge about the mechanisms behind the processes of *Haemonchus contortus* in order to help in tool production and to facilitate the reduction in anthelmintic resistance and promote the development of other control strategies, such as anthelmintic drug targets and vaccines [5]. **Phytotherapy.** Ocampo-Gutiérrez et al. observed that flavonol and flavonoid derivative compounds such as coumaric acid possess high nematocidal activity and they could be assessed in future studies searching for natural phytocompounds for controlling haemonchosis in small ruminants [6]. Komáromyová et al. studied available dietary antioxidants in the form of sainfoins [7]. They sustain the antibody response and thus indirectly improve the resistance of the animals against *H. contortus.* Springer et al. concluded that although rMSP alone did not lead to significant reduction in worm reproduction, it might still prove useful as part of a multi-component vaccine due to synergistic or additive effects [8]. **Management.** Bricarello et al. asserted that genetic selection to improve herd or flock parasite resistance to gastrointestinal nematode infection may also be incorporated into a holistic control plan, aiming at a substantial reduction in the use of anthelmintics and endectocides to make grazing systems more sustainable [9]. **Biological control.** Li et al. discussed the need to discover novel and high-efficiency nematicidal isolates and the application of our understanding to the appropriate selection of associated applications [10].

Although many of these organisms naturally have chemical molecules or secondary metabolites in their constitution, many of the solutions presented here are natural. The sub-products produced by these organisms could be used in the industry. However, these investments are scarce or uninteresting. Could it be that some levels of industry and certain entrepreneurs want to perpetuate the problem rather than solve it? Ideas, methods and processes are launched every day in research centres and universities, and that is the most important point. This Special Issue also features researchers from various countries and continents.
